# Economic burden and return on investment of immunization programs in Saudi Arabia: a health economic evaluation

**DOI:** 10.1186/s12889-026-28633-1

**Published:** 2026-07-20

**Authors:** Ali M. Alshahrani, Ahmed M. Ashour

**Affiliations:** 1 Department of Clinical Pharmacy, College of Pharmacy, Taif University, Taif, Saudi Arabia; 2 Department of Pharmacology and Toxicology, College of Pharmacy, Umm Al-Qura University, Makkah, Saudi Arabia

**Keywords:** Vaccine-preventable diseases, Immunization programs, Economic burden, Return on investment, Health economics

## Abstract

**Background:**

Vaccine-preventable diseases (VPDs) continue to impose a significant health and economic burden globally, despite advances in immunization programs. Narrower to the context of Saudi Arabia, the current literature consistently shows that the high vaccination coverage has had the primary impact of reducing disease incidence. Regardless, the broader economic impact of VPDs and the financial benefits of immunization remain important for policy evaluation within Saudi Arabia.

**Methods:**

This study employed a model-based economic evaluation using a societal perspective in order to carry out an estimation of the economic burden of measles, influenza, and pneumococcal diseases. We utilized the Cost of Illness (COI) approach for the purpose of quantifying direct medical costs and indirect productivity losses. On the other hand, the Value of Statistical Life (VSL) approach helped in the estimation of the monetary value of mortality reduction. A comparative framework analyzed current vaccination coverage against a counterfactual no-vaccination scenario for the calculation of the return on investment (ROI).

**Results:**

The estimated annual economic burden of the three selected VPDs in the absence of vaccination was USD 385 (95% CI: 315–460) million. Immunization programs generated substantial economic benefits, with total benefits estimated at USD 1085 (95% CI: 815–1360) million annually. The calculated ROI was 9.85 (95% CI: 6.8–11.3), essentially an indication that for each dollar invested in vaccination, there was multiple economic returns yielded. Sensitivity analyses confirmed the robustness of these findings.

**Conclusion:**

Immunization programs in Saudi Arabia provide significant economic and public health benefits and for this reason, sustained investment in vaccination is fundamentally essential towards the reduction of disease burden, improve population health, and ultimately support long-term economic productivity.

## Introduction

Vaccine-preventable diseases (VPDs) remain a significant public health challenge even with the decades of progress in immunization coverage and vaccine development for many of these ailments [1]. Diseases such as measles, influenza, pneumococcal infections, and rotavirus continue to contribute substantially to global morbidity and mortality, particularly in low- and middle-income countries [2–4]. While the clinical benefits of factors like vaccination and reduced disease incidence as well as complications and mortality are well established, current literature in this area points that there is a growing recognition that immunization programs also generate substantial economic benefits [4]. Efficiency in the allocation of the limited resources, the creation and implementation of robust policies for better health outcomes both call for the understanding of the economic burden of VPDs and the financial returns associated with vaccination as this is an essential avenue towards sustainable financing strategies in healthcare. Notably, in a recent study McLaughlin et al. content that the economic burden of VPDs extends beyond direct medical costs particularly as it encompasses a wide range of indirect and societal costs [5]. Direct costs include expenditures related to hospitalization, outpatient care, medications, and diagnostic services. Indirect costs, on the other hand, arise from productivity losses due to illness, disability, and premature mortality. In many settings, especially in low- and middle-income countries, these indirect costs can, inevitably, exceed direct healthcare expenditures which is a notably an occurrence that can place a substantial strain on households and national economies. Additionally, intangible costs such as reduced quality of life and long-term disability, while not easy to quantify, further amplify the overall burden. Beyond mortality and the associated challenges as out, vaccine-preventable diseases are also associated with substantial morbidity-related disability which includes temporary or long-term functional impairment that can affect areas like educational attainment and workforce participation or even the overall quality of life. These disability-related consequences are often underrepresented in economic evaluations despite contributing meaningfully to indirect costs and societal burden.

Immunization programs represent one of the most cost-effective public health interventions available to nations across the world. Through the preventing of diseases before they occur, vaccines help solve the critical problem of need for costly medical treatments and, from an economic point of view, mitigate productivity losses [6]. Over the past two decades, global initiatives, particularly those organized and led by institutions like the World Health Organization and Gavi, the Vaccine Alliance, have significantly expanded global access to vaccine more specifically within resource-limited settings [7]. These efforts have contributed to substantial declines in the incidence of several VPDs, demonstrating both health and economic gains. However, disparities in vaccine coverage persist, and outbreaks of preventable diseases continue to occur, underscoring the need for sustained investment in immunization programs. From an economic perspective, evaluating the value of vaccination requires robust methodological approaches that capture both costs and benefits over time. Traditional cost-effectiveness analyses have been widely used to assess health interventions, often reporting outcomes in terms of cost per disability-adjusted life year (DALY) averted [8]. While useful, these approaches may not fully capture the broader economic benefits of vaccination. More comprehensive frameworks, such as the Cost of Illness (COI) and Value of Statistical Life (VSL) approaches, have been increasingly employed to estimate the total economic impact of VPDs and the return on investment (ROI) of immunization programs. The COI approach quantifies the economic burden associated with disease, including direct and indirect costs, while the VSL approach assigns a monetary value to reductions in mortality risk, reflecting societal willingness to pay for health improvements.

Saudi Arabia has established a comprehensive national immunization program that is focused on providing routine childhood and selected adult vaccinations free of charge through the Ministry of Health as part of its commitment to Universal Health Coverage and Vision 2030. The program has achieved consistently high vaccination coverage for many routine childhood vaccines and has substantially reduced the incidence of several vaccine-preventable diseases. Nevertheless, seasonal influenza, pneumococcal disease, and occasional measles outbreaks continue to impose preventable clinical and economic burdens, particularly among high-risk populations [9]. As healthcare expenditure continues to increase, evaluating the economic value generated by immunization programs is inevitably important for supporting evidence-based resource allocation and long-term investment decisions.

Recent advances in vaccine economics, including methodologies developed through initiatives such as the Decade of Vaccine Economics (DOVE) project, provide structured frameworks for estimating the ROI of immunization programs [10]. These methodologies enable researchers and policymakers to compare the costs of vaccination with the economic benefits derived from disease prevention under counterfactual scenarios where no vaccination occurs. Such analyses are particularly valuable in guiding investment decisions, especially in settings with constrained healthcare budgets. Despite the availability of these analytical tools, there remains a need for comprehensive assessments that integrate both the economic burden of VPDs and the financial benefits of immunization programs within a unified framework. Despite the availability of these analytical tools, evidence from Saudi Arabia remains limited. Existing economic evaluations have largely examined individual vaccines or specific disease outcomes whereas comprehensive assessments integrating disease burden, Cost of Illness, Value of Statistical Life, and Return on Investment within a unified analytical framework are scarce. Consequently, local evidence to support vaccine investment priorities and economic decision-making remains limited. Furthermore, variations in methodology, data sources, and assumptions can lead to inconsistencies in reported economic outcomes [10–12]. Addressing these gaps is essential for strengthening the evidence base and supporting more informed decision-making in public health policy.

From the disease of focus perspective, the current study evaluated measles, influenza, and pneumococcal disease because these vaccine-preventable diseases collectively represent diverse epidemiological and economic profiles within Saudi Arabia [13–15]. Recent findings by Alruqaie et al. show that measles remains a highly transmissible disease with outbreak potential despite high vaccination coverage [14]. In addition, iinfluenza causes substantial annual morbidity and productivity losses, and pneumococcal disease is associated with considerable mortality and healthcare utilization, particularly among young children, older adults, and individuals with chronic medical conditions [15]. Together, these diseases capture acute epidemic risk, recurring seasonal disease burden, and severe invasive bacterial infections, thereby providing a comprehensive assessment of the economic value of immunization across different public health contexts.

This study aimed to evaluate the economic burden and return on investment of immunization against measles, influenza, and pneumococcal disease in Saudi Arabia using integrated Cost of Illness and Value of Statistical Life approaches. These three diseases were selected because of their continued public health relevance, differing epidemiological characteristics, and substantial healthcare and societal costs despite the availability of effective vaccines. By integrating COI and VSL within a unified ROI framework, this study provides locally relevant evidence to inform immunization policy, resource allocation, and future vaccine investment decisions in Saudi Arabia. In particular, these methods allowed for the estimation of both the direct and indirect costs associated with the three selected VPDs of focus and the economic returns generated by vaccination on the same. This study is among the first in Saudi Arabia to integrate COI and VSL approaches within a unified ROI framework.

## Methodology

### Study design

This study employed a model-based economic evaluation to estimate the economic burden of three selected vaccine-preventable diseases and also to assess the financial benefits of immunization programs in Saudi Arabia. We employed a comparative analytical framework in which we incorporated two scenarios as follows; one, the vaccination scenario which reflected existing immunization coverage, and two, a counterfactual scenario under which we assumed no vaccination and which approach allowed for the estimation of the return on investment (ROI) associated with national immunization efforts within the country. This economic evaluation was reported in accordance with the Consolidated Health Economic Evaluation Reporting Standards (CHEERS 2022) statement. The CHEERS checklist was used to guide the reporting of study design, model assumptions, data sources, costing methods, outcome valuation, uncertainty analysis, and interpretation of findings.

### Study perspective and diseases selection

We carried out the analysis from a societal perspective, essentially capturing both direct healthcare costs and indirect, particularly productivity losses due to morbidity and premature mortality. In the analysis, we adopted a long-term time horizon to capture both short- and long-term economic and health outcomes. As earlier indicated and for emphasis, in this study we focused on three high-burden VPDs relevant to the Saudi context namely measles, influenza, and pneumococcal disease. We arrived at the choice for these particular VPDs diseases on the basis of their epidemiological relevance, inclusion in national immunization programs, and availability of relevant data to enable us carry out the analysis and attain meaningful conclusions. The analysis was conducted from a societal perspective and therefore included the population eligible to receive immunization services within Saudi Arabia, irrespective of nationality. Epidemiological and economic inputs were derived from national population estimates without separate disaggregation of Saudi citizens and expatriate residents because publicly available aggregate data do not consistently distinguish these populations for all model parameters. Age-specific stratification was incorporated where data were available to reflect differences in disease burden and vaccine impact.

### Health economic analysis plan

The study was conducted as a model-based economic evaluation from a societal perspective. The analysis compared current vaccination coverage in Saudi Arabia with a hypothetical no-vaccination scenario. Economic outcomes included direct medical costs, indirect productivity losses, mortality-related economic losses, total economic burden, and return on investment (ROI). All costs were standardized to 2024 United States Dollars (USD). A discount rate of 3% was applied to future productivity losses and mortality-related benefits, consistent with international health economic evaluation recommendations.

### Currency and price year

All costs were reported in 2024 United States Dollars (USD). Historical cost estimates obtained from published literature were adjusted to 2024 values using inflation indices and purchasing power parity (PPP) conversion factors where applicable.

### Data sources and model inputs

Epidemiological data were obtained from publicly available sources including the World Health Organization (WHO), Saudi Ministry of Health (MOH), UNICEF, and World Bank databases. Model parameters included disease incidence, mortality rates, vaccination coverage, treatment costs, productivity losses, labor force participation rates, life expectancy, and gross domestic product (GDP) per capita.

When Saudi-specific cost estimates were unavailable, values from Gulf Cooperation Council (GCC) countries and comparable Middle Eastern healthcare systems were used. All transferred estimates were adjusted using purchasing power parity (PPP) and inflation conversion methods. Parameter ranges were derived from published literature and incorporated into sensitivity analyses. To ensure data accuracy, transparency, consistency and reliability, we cross-checked all data inputs across multiple national and international sources wherever possible. Preference was given to Saudi Ministry of Health and other national datasets, while international sources such as WHO, UNICEF, and the World Bank were used to supplement unavailable local parameters. Where model inputs were derived from published literature, values were selected based on methodological quality, relevance to the Saudi context, and consistency with previous economic evaluations. The principal model parameters, base-case estimates, ranges used in sensitivity analyses, and data sources are summarized in Table 1.

**Table 1:** Key model parameters and data sources

Parameter	Base-Case Estimate	Range Used in Sensitivity Analysis	Source
Measles annual cases (no-vaccination scenario)	20,000	15,000–25,000	WHO data and model estimates [13, 14]
Influenza annual cases (no-vaccination scenario)	1,500,000	1,200,000–1,800,000	WHO/MOH data and model estimates
Pneumococcal disease annual cases (no-vaccination scenario)	55,000	40,000–70,000	WHO data and model estimates
Measles economic burden	USD 45 million	USD 40–60 million	Model estimates
Influenza economic burden	USD 185 million	USD 150–220 million	Model estimates
Pneumococcal disease economic burden	USD 150 million	USD 120–180 million	Model estimates
Vaccination coverage	> 90%	85–95%	WHO [13], UNICEF, MOH
Immunization program costs	USD 100 million	USD 80–120 million	Model estimates and literature
Value of Statistical Life (VSL)	USD 4 million	USD 3–5 million	Literature-based income-adjusted estimates [16–18]
Total economic benefits	USD 1,085 million	USD 815–1,360 million	Model estimates
Discount rate	3%	0–5%	Health economic evaluation guidelines

### Model assumptions

Several assumptions were necessary for model development:

1. Current vaccination coverage reflects routine immunization performance in Saudi Arabia.
2. Disease incidence under the no-vaccination scenario was estimated by combining published pre-vaccination epidemiological evidence, current national surveillance data, vaccine coverage estimates, and published vaccine effectiveness estimates to approximate the expected disease burden in the absence of immunization.
3. Productivity losses were estimated using the human capital approach.
4. Future earnings were discounted at 3% annually.
5. Regional healthcare cost estimates were assumed transferable after economic adjustment.
6. VSL estimates were transferred using internationally accepted income-adjustment methods.

These assumptions were evaluated through sensitivity analyses.

### Estimation of economic burden

The economic burden of VPDs was estimated using the Cost of Illness (COI) approach, incorporating both direct and indirect costs. For costs, direct medical costs included expenditures related to hospitalization, outpatient care, pharmaceuticals, and diagnostic services. These costs were estimated by multiplying disease-specific unit costs (sourced from literature) by the number of reported cases in Saudi Arabia. Indirect costs were calculated using the human capital approach. In occurrences where disease-specific unit cost estimates varied across sources the estimation process entailed giving preference to studies conducted in Saudi Arabia or neighboring GCC countries. From these variables, we used the model equation below to estimate COI:

$$\mathrm{COI}=\text{Direct Costs} + \text{Indirect Costs}$$

In this model the two main inputs were the

$$\begin{aligned}&\text{Direct Costs} =\sum\left(\text{Number of Cases}\times\text{Unit Medical Cost}\right)\mathrm{and}\\& \text{Indirect Costs = Productivity Losses Due to Morbidity}\\& + \text{Productivity Losses Due to Premature Mortality}\end{aligned}$$

Productivity losses due to morbidity were estimated based on workdays lost and average wage rates while mortality-related productivity losses were estimated using the present value of future earnings approach. In addition, for all diseases lacking locally reported treatment costs we utilized regional averages following currency normalization and healthcare expenditure adjustment procedures. Through this approach, we were in a position to improve regional applicability while acknowledging limitations in country-specific cost reporting. Productivity losses due to morbidity were estimated based on average days of work lost per case and mean wage rates. For premature mortality, the present value of future earnings was calculated using life expectancy data and national income indicators. The total economic burden under the no-vaccination scenario was computed as the sum of direct and indirect costs across all selected diseases. All analyses and model calculations were performed using Microsoft Excel 365 (Microsoft Corp., Redmond, WA, USA).

### Valuation of mortality risk and estimation of immunization cost programs

To estimate the economic value of mortality reduction, the Value of Statistical Life approach was applied. Given the absence of country-specific VSL estimates for Saudi Arabia, baseline VSL values from international literature were adjusted using income elasticity and GDP per capita data from the World Bank. We adopted this income-adjustment approach because in many past studies, it has been widely applied in health economic evaluations involving countries where directly estimated VSL data are unavailable. Sensitivity analyses using alternative elasticity assumptions were additionally conducted to account for uncertainty surrounding transferred VSL estimates. The number of deaths averted through vaccination was multiplied by the adjusted VSL for the purpose of estimating the monetary value of lives saved. The estimation model used for VSL was:

Adjusted VSL estimates for Saudi Arabia were calculated using an income-adjustment transfer approach based on the following equation:

$$\mathrm{VSL}\_\mathrm{SA}=\mathrm{VSL}\_\mathrm{reference}\times\left(\mathrm{Income}\_\mathrm{SA}/\mathrm{Income}\_\mathrm{reference}\right)^\beta$$

With VSL_SA representing the adjusted Value of Statistical Life for Saudi Arabia, VSL_reference represents the baseline VSL obtained from published international literature, Income_SA represents Saudi Arabia’s GDP per capita, Income_reference represents GDP per capita of the reference country, and β represents the income elasticity parameter.

The total monetary value of deaths averted was then estimated as:

$$\begin{aligned}&\text{Monetary Value of Deaths Averted = Number of Deaths Prevented}\\&\times\text{Adjusted VSL}\_\mathrm{SA}\end{aligned}$$

### Double counting avoidance

Important to point out here is that in order to address potential concerns regarding overlapping valuation components we applied two complementary economic valuation approaches. The cost-of-illness framework was used to estimate direct medical costs and productivity losses avoided due to vaccination as described above and then, mortality benefits were valued separately using the Value of Statistical Life approach. COI-based mortality-related productivity losses were excluded from the VSL-based scenario to avoid potential overlap in valuation components. This dual-framework approach allows for comparison of conservative (COI-based) and broader societal (VSL-based) benefit estimates.

### Return on investment (ROI) calculation

The ROI of immunization programs was calculated by comparing total economic benefits with total program costs. Economic benefits included one, the averted direct and indirect costs (COI), and two, the monetary value of deaths averted (VSL). ROI was calculated using the following formula:

$$\begin{aligned}&\text{ROI = }\left(\text{Total Economic Benefits}-\text{Total Immunization Costs}\right)\\& / \text{Total Immunization Costs}\end{aligned}$$

Important to note, the reported ROI values were calculated using unrounded model outputs. Accordingly, manual calculations based on rounded summary values presented in the Results may differ slightly due to rounding.

### Characterization of heterogeneity

Where data permitted, disease burden and vaccination impact were assessed separately across pediatric and adult populations. Differences in disease incidence, mortality risk, and productivity losses between age groups were considered in model estimates. However, limited availability of age-stratified cost data prevented full subgroup economic analyses.

### Distributional effects

Although the primary analysis focused on aggregate economic outcomes, immunization programs are expected to generate important equity benefits through disease prevention among vulnerable populations, including children, older adults, and individuals with chronic illnesses. Distributional effects were not quantitatively modeled and therefore may underestimate the broader societal value of vaccination.

### Uncertainty analysis

One-way sensitivity analyses were conducted on key model parameters including disease incidence, treatment costs, productivity losses, discount rates, vaccination program costs, and Value of Statistical Life (VSL) estimates. Parameter ranges were based on published literature and expert assumptions. Additional scenario analyses examined alternative assumptions regarding vaccination coverage, mortality reduction, and income elasticity used for VSL transfer. Results are presented as 95% uncertainty intervals. Uncertainty intervals were generated by recalculating model outputs using the lower and upper bounds of each parameter specified in the one-way sensitivity analyses; therefore, these intervals represent deterministic uncertainty ranges rather than probabilistic confidence intervals. Probabilistic sensitivity analysis was not undertaken because joint probability distributions for several key Saudi-specific model parameters were unavailable. Accordingly, deterministic one-way and scenario analyses were considered the most appropriate approach for evaluating parameter uncertainty. The purpose of these analyses was to evaluate the robustness of conclusions under plausible alternative assumptions.

## Results

### Epidemiological overview

Data obtained from the World Health Organization and the Saudi Ministry of Health indicated that vaccination coverage for key childhood immunizations in Saudi Arabia exceeded 90% in recent years, particularly for measles-containing vaccines. Despite high coverage, sporadic cases of measles continued to be reported, primarily due to gaps in herd immunity and imported infections. Seasonal influenza remained a significant contributor to morbidity, particularly among elderly and high-risk populations, while pneumococcal disease continued to impose a measurable burden in both paediatric and adult populations. Under the counterfactual no-vaccination scenario, the estimated incidence of these diseases increased substantially. Measles cases were projected to rise sharply due to its high transmissibility, while influenza and pneumococcal disease showed moderate but consistent increases in incidence and associated complications.

Using the Cost of Illness (COI) approach, the total economic burden of the selected VPDs in the absence of vaccination was estimated to be substantial. The total annual economic burden for measles was estimated at approximately USD 45 million (95% CI: 40–60), driven primarily by hospitalization costs and productivity losses associated with parental caregiving and complications in children. Influenza accounted for the largest share of economic burden, estimated between USD 185 million (95% CI: 150–220) annually. This was largely attributable to outpatient visits, hospitalizations among high-risk groups, and significant productivity losses due to absenteeism. Lastly, the economic burden pneumococcal disease was estimated at USD 150 million (95% CI: 120–180) annually, essentially an indication of high treatment costs for severe cases such as pneumonia and meningitis, as well as mortality-related productivity losses. The total combined economic burden for these three diseases was USD 385 (95% CI: 315–460). Table 2 provides the summary of these estimates.

**Table 2:** Estimated economic burden of selected vaccine-preventable diseases under no vaccination scenario

Disease	Estimated Cases (Annual)	Direct Costs (USD Millions)	Indirect Costs (USD Millions)	Total Economic Burden (USD Millions, 95% CI)
Measles	15,000–25,000	20–25	25–35	45 (95% CI: 45–60)
Influenza	1,200,000–1,800,000	80–100	70–120	185 (95% CI: 150–220)
Pneumococcal Disease	40,000–70,000	60–90	60–90	150 (95% CI: 120–180)
Total	-	160–215	155–245	385 (95% CI: 315–460)

### Indirect costs and productivity losses and value of mortality risk reduction

Indirect costs constituted a significant proportion of the total economic burden, accounting for an estimated 40–60% of total costs across the three diseases (see Table 2). Productivity losses due to missed workdays were particularly pronounced for influenza, given its high incidence among working-age adults. In contrast, pneumococcal disease and measles contributed more heavily to mortality-related productivity losses, particularly in vulnerable populations.

Application of the Value of Statistical Life (VSL) approach demonstrated substantial economic benefits associated with mortality reduction. Adjusted VSL estimates for Saudi Arabia, based on income scaling from global benchmarks, ranged between USD 3 million and 5 million per statistical life. The estimated number of deaths averted annually through immunization resulted in a total economic benefit ranging from USD 500 million to 900 million. Pneumococcal vaccination contributed the largest share of mortality-related economic benefits due to its association with severe outcomes in both children and older adults.

### Cost of immunization programs and return on investment (ROI)

The total annual cost of immunization programs targeting the selected diseases in Saudi Arabia was estimated to range between USD 80 and 120 million. These costs included vaccine procurement, distribution, cold chain maintenance, and administration. Childhood vaccination programs accounted for the majority of expenditures, while seasonal influenza vaccination contributed a smaller but still significant portion.

Through a combination of averted treatment costs and the economic value of lives saved, we estimated the total economic benefits of immunization programs was USD 1085 million (95% CI: 815–1360) annually and using these estimates, the ROI for immunization programs in Saudi Arabia estimated to be 9.85 (95% CI: 6.8–11.3) (Table 3). These findings indicated that immunization programs substantially reduced disease morbidity and mortality while generating considerable economic returns. For every USD 1 invested in immunization, approximately USD 6.8–11.3 in economic benefits were generated through avoided healthcare costs, reduced productivity losses, and the monetary value of deaths averted. A summary of the estimated economic benefits, immunization program costs, and return on investment is presented in Table 3.

**Table 3:** Economic benefits and return on investment (ROI) of immunization programs

Parameter	Mean Estimate	95% CI
Averted Treatment Costs (USD Millions)	385	315–460
Value of Lives Saved (USD Millions)	700	500–900
Total Economic Benefits (USD Millions)	1,085	815–1,360
Immunization Program Costs (USD Millions)	100	80–120
Return on Investment (ROI)	9.85	6.8–11.3

### Sensitivity analysis

Sensitivity analyses demonstrated that the model findings were robust across all plausible parameter ranges. Variations in disease incidence, treatment costs, vaccination program costs, discount rates, and VSL estimates affected the magnitude of the estimated economic burden and ROI but did not alter the overall conclusion that immunization programs generated substantial net economic benefits. The ROI remained consistently above 5 in all tested scenarios, with VSL estimates and disease incidence contributing the greatest influence on model outputs. These findings support the stability of the conclusions despite uncertainty surrounding individual model parameters.

## Discussion

The findings of this study demonstrated that vaccine-preventable diseases (VPDs) continue to impose a substantial economic burden in Saudi Arabia, despite the country’s high immunization coverage. By integrating Cost of Illness (COI) and Value of Statistical Life (VSL) approaches, the analysis provided a comprehensive assessment of both the direct and indirect costs associated with measles, influenza, and pneumococcal disease, as well as the significant financial returns generated through immunization programs [16–18]. Overall, the results reinforced the position that vaccination is not only a critical public health intervention but also a highly efficient economic investment [19]. One of the most notable findings was the disproportionately high economic burden associated with influenza compared to measles and pneumococcal disease. This result can be attributed to influenza’s high incidence rate, particularly among working-age adults, which translated into substantial productivity losses. Although influenza-related mortality was lower than that of pneumococcal disease, its widespread transmission resulted in a greater cumulative economic impact. This finding aligns with existing literature indicating that diseases with high incidence but relatively low mortality can still generate significant economic costs due to absenteeism and reduced workforce productivity.

In contrast, pneumococcal disease contributed more heavily to mortality-related costs, as reflected in the VSL estimates. Severe manifestations such as pneumonia and meningitis were associated with higher case fatality rates, particularly among young children and older adults. As a result, pneumococcal vaccination yielded substantial economic benefits through the prevention of premature deaths. Measles, while less economically burdensome in absolute terms due to lower incidence in Saudi Arabia, remained a critical concern due to its high transmissibility and potential for outbreaks in the absence of sustained immunization coverage. The findings highlighted the importance of maintaining high vaccination rates to prevent resurgence, especially in the context of global travel and population mobility. The estimated return on investment (ROI), ranging from approximately 6.8 to 11.3, underscored the strong economic value of immunization programs in Saudi Arabia. These results were consistent with global evidence reported by organizations such as the World Health Organization and Gavi, the Vaccine Alliance, which have documented high economic returns on vaccination across diverse settings. The findings demonstrated that investments in immunization not only reduce healthcare expenditures but also generate broader societal benefits by preserving productivity and preventing premature mortality [20–22].

In addition to direct medical costs and mortality-related losses the findings of the current study need to also be interpreted in the context of disability-related health loss associated with vaccine-preventable diseases. Morbidity episodes particularly those requiring hospitalization or prolonged recovery have the potential to result in temporary or persistent functional limitations that reduce workforce participation and productivity. Although not explicitly modeled as disability-adjusted life years in the current analysis, these effects are partially captured through indirect cost estimates an outcome which here is interpreted to mean that the true societal burden may be even higher than reported.

Several limitations should be considered when interpreting the findings. The analysis relied partially on secondary data and, in some cases, regional or global estimates adjusted to the Saudi context due to limited availability of country-specific cost data. While these adjustments were made using established methods, they may introduce uncertainty into the estimates. Also, the study assumed average cost and incidence values, which may not fully capture variations across different regions or population groups within Saudi Arabia. Further, intangible costs such as quality of life losses were not explicitly quantified, potentially leading to an underestimation of the total economic burden [20, 21]. In accordance with CHEERS 2022 recommendations, several sources of uncertainty should be acknowledged. The analysis relied on secondary data sources and transferred economic parameters due to limited availability of Saudi-specific estimates. Consequently, the reported estimates should be interpreted as indicative of the likely economic value of immunization rather than precise predictions of future healthcare expenditure or societal benefit. Furthermore, probabilistic uncertainty analysis was not performed. Consequently, the reported economic burden and ROI estimates should be interpreted as policy-oriented approximations rather than precise forecasts. The analysis was restricted to measles, influenza, and pneumococcal disease. Consequently, the overall economic value of Saudi Arabia’s national immunization programme is likely underestimated because several other vaccine-preventable diseases were not included. In addition, indirect effects of vaccination, including herd immunity and changes in disease transmission dynamics, were not explicitly modelled. Their inclusion may have resulted in greater estimated health and economic benefits.

Despite the limitations, the findings provide policy-relevant evidence supporting the economic value programs. The consistent observation of favorable ROI across deterministic sensitivity analyses supports the robustness of the findings, although the estimates should be interpreted in light of the underlying model assumptions. Future research could build on this work by incorporating more granular, country-specific data and expanding the analysis to include additional vaccine-preventable diseases and broader societal impacts. Notably immunization programs in Saudi Arabia deliver substantial economic and public health benefits [23–25]. Through the preventing disease, reducing healthcare costs, and preserving productivity, vaccines represent a cornerstone of both health policy and economic strategy [26–28]. Continued commitment to immunization will be essential for sustaining these gains and addressing emerging public health challenges.

### Implications of the findings

The findings carry important implications for health policy and resource allocation in Saudi Arabia. Sustained investment in immunization programs remains essential to maintaining current health gains and preventing the resurgence of highly transmissible diseases such as measles. In addition, expanding vaccination coverage for diseases such as influenza, particularly among high-risk and working-age populations, could further enhance both health and economic outcomes. Policymakers should therefore continue to prioritize immunization as a central component of national health strategy, supported by strong surveillance systems and evidence-based decision-making. While the study provided robust estimates, it also highlighted the need for improved availability of country-specific economic and epidemiological data to enhance the precision of future analyses. Further research incorporating more granular data and additional vaccine-preventable diseases would strengthen the evidence base and support more targeted policy interventions. Ultimately, immunization programs in Saudi Arabia not only protect population health but also generate substantial economic value. By reducing disease burden, lowering healthcare costs, and supporting economic productivity, vaccines represent a critical investment in both public health and national development. Continued commitment to immunization will be essential for sustaining these benefits and addressing future health challenges.

## Conclusion

This study demonstrated that vaccine-preventable diseases (VPDs) continue to impose a measurable economic burden in Saudi Arabia, even within a context of relatively high immunization coverage. Through the applying of both the Cost of Illness (COI) and Value of Statistical Life (VSL) approaches, the analysis provided a comprehensive assessment of the direct, indirect, and mortality-related costs associated with measles, influenza, and pneumococcal disease. The study suggests that immunization programmes generate substantial economic benefits. The findings highlighted that, in the absence of vaccination, these diseases would generate substantial healthcare expenditures and productivity losses, placing a significant strain on both the healthcare system and the broader economy. At the same time, the study clearly demonstrated that immunization programs yield considerable economic benefits. The estimated return on investment (ROI), ranging from approximately 6.8 to 11.3, indicated that vaccination programs generated multiple times their initial cost in economic returns. These benefits were driven not only by reductions in treatment costs but also by the prevention of premature mortality and the preservation of workforce productivity. The results reinforced the position that immunization is one of the most cost-effective and economically valuable public health interventions available.

## Data Availability

All data used in this study were obtained from publicly available sources, including the World Health Organization (WHO), World Bank, UNICEF, and Saudi Ministry of Health databases. The datasets analyzed during the current study are available from the respective public repositories cited in the manuscript. No individual-level or confidential data were used.
